# Use of Condition-Specific Patient-Reported Outcome Measures in Clinical Trials among Patients with Wrist Osteoarthritis: A Systematic Review

**DOI:** 10.1155/2012/273421

**Published:** 2012-11-01

**Authors:** Steven M. McPhail, Karl S. Bagraith, Mandy Schippers, Paula J. Wells, Anna Hatton

**Affiliations:** ^1^Centre for Functioning and Health Research, Queensland Health, Cnr of Ipswich Road and Cornwall Street, Buranda, Brisbane, QLD 4102, Australia; ^2^School of Public Health and Institute of Health and Biomedical Innovation, Queensland University of Technology, Victoria Park Road, Kelvin Grove, Brisbane, QLD 4059, Australia; ^3^Occupational Therapy Department and Centre for Allied Health Research, Royal Brisbane and Women's Hospital, Butterfield Street, Herston, Brisbane, QLD 4029, Australia; ^4^School of Health and Rehabilitation Sciences, The University of Queensland, Services Road, St Lucia, Brisbane, QLD 4072, Australia

## Abstract

*Background*. This paper aimed to identify condition-specific patient-reported outcome measures used in clinical trials among people with wrist osteoarthritis and summarise empirical peer-reviewed evidence supporting their reliability, validity, and responsiveness to change. *Methods*. A systematic review of randomised controlled trials among people with wrist osteoarthritis was undertaken. Studies reporting reliability, validity, or responsiveness were identified using a systematic reverse citation trail audit procedure. Psychometric properties of the instruments were examined against predefined criteria and summarised. *Results*. Thirteen clinical trials met inclusion criteria. The most common patient-reported outcome was the disabilities of the arm, shoulder, and hand questionnaire (DASH). The DASH, the Michigan Hand Outcomes Questionnaire (MHQ), the Patient Evaluation Measure (PEM), and the Patient-Reported Wrist Evaluation (PRWE) had evidence supporting their reliability, validity, and responsiveness. A post-hoc review of excluded studies revealed the AUSCAN Osteoarthritis Hand Index as another suitable instrument that had favourable reliability, validity, and responsiveness. *Conclusions.* The DASH, MHQ, and AUSCAN Osteoarthritis Hand Index instruments were supported by the most favourable empirical evidence for validity, reliability, and responsiveness. The PEM and PRWE also had favourable empirical evidence reported for these elements. Further psychometric testing of these instruments among people with wrist osteoarthritis is warranted.

## 1. Background 

Wrist osteoarthritis is a common condition treated by upper limb orthopaedic teams [1]. Clinical trials evaluating new advances and ongoing refinement of intervention approaches (both surgical and nonsurgical) for this clinical group require the use of appropriate outcome measures to determine their effect. Objective evaluation approaches such as radiographic investigations and other measures of body function and structure are central to this process [2]. However, patient-reported outcomes consisting of questionnaire or rating scale approaches are a valuable method of gaining quantitative information about the lived experiences of people with the condition [3]. Longitudinal use of patient-reported outcomes over multiple assessment points can permit comparisons of pain, function, and health-related quality of life within an individual over time, as well as comparisons between groups of participants in clinical trials. 

The use of patient-reported outcomes as primary measures in clinical trials has become increasingly popular over recent decades [4]. There are now multiple patient-reported outcomes available for use in clinical trials for most orthopaedic conditions [5]. Some instruments are generic in nature and include questions designed to summarise perceived health in relation to broad life domains [3]. These instruments are designed for use across a wide range of clinical groups [3]. Two examples of generic instruments include the Medical Outcomes Study 36-Item Short-Form Health Survey (SF-36) and EuroQol-5D (EQ-5D) [6, 7]. There has been considerable empirical research reporting favourable measurement properties of these generic instruments [8–21]. In summary, generic health-related quality of life instruments capture multiple aspects of health-related quality of life and can be particularly useful for making comparisons of patient-reported health states across groups with heterogeneous clinical conditions [9, 12, 14, 15, 17, 18, 20, 22]. Pain scales are another group of patient-reported outcome measures that have been widely used [23]. Comprehensive critical analyses of pain evaluation approaches have been reported previously [23–28]. In summary, there is evidence to support the use of pain scales across a broad range of conditions in clinical trials [23, 24, 26–28]. However, amongst wrist osteoarthritis patients, the effects of the condition extend beyond discomfort into areas of impairment and activity limitation that can impact several key areas of functioning and health-related quality of life.

The use of condition-specific instruments has great potential for evaluating domains of physical functioning and health-related quality of life commonly affected by a specific pathology or body region dysfunction [22]. Condition-specific instruments include questions focused on issues or aspects of health that are commonly affected by the specific condition or body region dysfunction that is under consideration [3]. In the context of wrist osteoarthritis, condition-specific patient-reported outcome measures can be used to evaluate the impact of osteoarthritis on the upper limb, wrist and hand functioning. Although the content of condition-specific instruments may vary somewhat depending on whether they focus on pathological symptoms or body region functioning (or both), they share a common goal of evaluating key elements of disability or health-related quality of life relevant to wrist arthritis sufferers [29]. This specific focus may enable these instruments to better reflect changes relevant to wrist arthritis patients during clinical trials evaluating the effects of targeted interventions [29, 30]. Similarly, it may reduce the chance of a treatment effect being diluted among other health-related influences (either positive or negative) that are not relevant to the wrist osteoarthritis intervention under consideration during randomised trials of clinical efficacy [30–32]. 

The selection of patient-reported outcomes for use in clinical trials should be informed by empirical research supporting several key instrument properties [33]. It is critical that outcome measures used in clinical trials are reliable and valid [3, 33–35]. Only guarded conclusions (at best) can be drawn from patient assessments when uncertainty exists regarding the reliability or validity of an assessment instrument [3]. Additionally, it is also important that patient-reported outcomes are responsive to change [36–38]. An instrument that is not responsive to change is likely to increase the chance of a false negative finding during a clinical trial [39]. Such a finding would not only confound the results of the clinical trial at hand, but potentially stifle future research in the field. 

Prior critical analyses examining the psychometric or clinimetric properties of outcome measures with relevance to orthopaedic upper limb patients have been undertaken [33–35, 40, 41]. These reviews have included objective physical or observational measures as well as patient-reported outcomes evaluating symptoms, functioning, and participation in daily activities across heterogeneous clinical groups. A systematic review of disability measures for use in a population-based survey of people with hand osteoarthritis has also been reported [42]. However, there has been no prior review of condition-specific patient-reported outcomes used in clinical trials amongst patients with wrist osteoarthritis. The nature of clinical interventions for wrist osteoarthritis differs from other common conditions affecting the wrist or hand (such as rheumatoid arthritis or acute tendon injuries). The purpose of this paper is to report a systematic review examining which condition-specific (or body region specific) patient-reported outcomes have been used in clinical trials among people with wrist osteoarthritis. This paper will identify which instruments have been used and examine whether they have evidence to support their validity, reliability, and responsiveness. It is intended that this information will be useful for informing instrument selection in future clinical trials and highlighting priorities for future research in the field. 

The specific aims of this paper are therefore threefold: first, to identify which condition-specific patient-reported outcome measures have been used during randomised controlled trials among people with wrist osteoarthritis; second, to examine whether these instruments are supported by peer-reviewed published empirical data reporting their reliability, validity, or responsiveness, third, to discuss priorities for future research to improve the quality of condition-specific patient-reported outcomes for use in clinical trials. 

## 2. Methods

### 2.1. Design

A systematic review of condition-specific patient-reported outcome measures used in randomised trials among people with wrist osteoarthritis was undertaken.

### 2.2. Search Strategy

**Figure 1:**
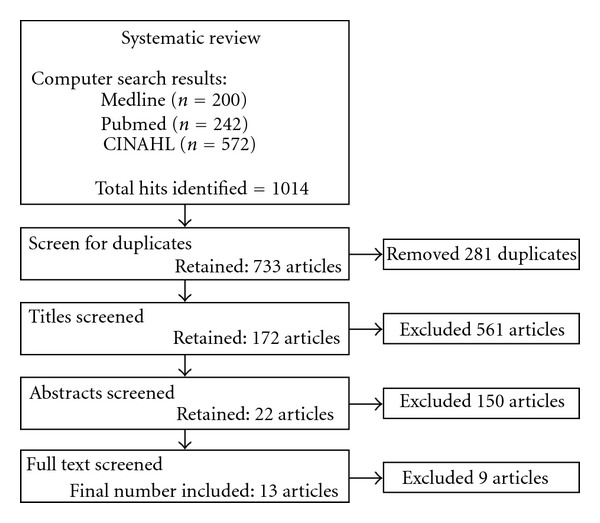
Search results and manuscripts excluded at each of the four stages.

Searches were performed in Medline, National Institutes of Health online database, PubMed, and CINAHL from the earliest records until the date of the search (January 2012). Search terms included combinations of terms (“wrist” or “carpal” or “radiocarpal”) and (“arthritis” or “osteoarthritis”) and (“trial” or “RCT”). These terms were used as keywords and expanded Medical Subject Headings (MESH) terms to search all text. A conventional four-stage screening approach to identify studies meeting the inclusion and exclusion criteria was undertaken by two researchers (Figure 1). A third member of the research team was available to arbitrate any disagreement between the two researchers but was not required. During the first stage duplicates were removed. During the second stage, studies were screened by title and non-relevant articles were removed. During the third and fourth stages, articles were screened by abstract and full text respectively, to examine whether articles met the criteria of exclusion or inclusion with non-relevant articles removed at each stage (Figure 1). 

### 2.3. Study Selection

**Inclusion and Exclusion**

Manuscripts reporting randomised clinical trials among adults with wrist osteoarthritis were included. This included samples with arthritis affecting any joint adjacent to the carpal bones (including trapeziom-metacarpal joint). Studies that included participants with osteoarthritis of other (non-carpal) joints in the hand or elsewhere in the body were excluded. Studies among clinical groups with other inflammatory arthritis such as rheumatoid arthritis or juvenile arthritis were excluded. Manuscripts that did not specify the type of arthritis present in their sample were also excluded. There were no exclusion criteria based on the intervention type or length of follow-up within the trial as these factors were not under consideration in this paper.

### 2.4. Data Extraction and Instrument Property Assessment

The population, interventions, and all outcome measures used in each of the included studies were summarised and tabulated (Table 1). Condition-specific patient-reported outcome measures were identified and tabulated (Table 2). A reverse citation trail audit was then undertaken to identify published peer reviewed literature describing attributes of reliability, validity, and responsiveness for each of the patient-reported outcomes. This process involved identifying the publication (or publications) in which the patient-reported outcome was first reported (or the publication which reported the appropriate version). This was determined through following the trail of citations for each instrument included in the arthritis trials, back to the primary source reporting the instrument. We termed this article the “primary reference.” The authors considered it likely that studies reporting reliability, validity, and responsiveness for each of the instruments would have cited this primary instrument publication. Citations for each primary reference listed in Scopus, Pubmed, and Google Scholar were then manually reviewed to determine which studies reported components of reliability, validity, and responsiveness. 

A summary of the elements of validity, reliability, and responsiveness that have been reported for each of the instruments were then tabulated (Table 3). Psychometric evaluation of the identified measures was guided by established criteria [63, 64], which are widely employed to assess the quality of patient-reported outcome measures [65, 66]. With respect to the aims of this study, we considered evidence for content validity, construct validity, internal consistency, agreement (absolute measurement error between repeated measures expressed in unit of measurement of the scale), reliability (reliability coefficients expressed as a ratio between 0 and 1), and responsiveness of each measure. The evidence was rated against predetermined criteria and classified into four ratings (details of which are provided in the aforementioned studies [63, 64], and a brief description of ratings is also included below Table 3). To investigate whether less strict study exclusion criteria would have resulted in the inclusion of other high quality instruments (in terms of validity, reliability, and responsiveness) a post-hoc examination of outcome measures from studies excluded on the basis of containing mixed diagnoses samples (rheumatoid arthritis and wrist osteoarthritis) was also undertaken. 

## 3. Results 

### 3.1. Search Results

The searches returned a total of 1014 hits (Figure 1). This included 733 unique articles after the removal of 281 duplicates. Screening by title then abstracts removed a further 561 and 150 articles, respectively. Full texts of the remaining 22 manuscripts were then retrieved and a further 9 studies were excluded. Thirteen clinical trials met all criteria and were included in the paper. Details of the sample, the intervention under investigation, and the outcome measures used in each of the thirteen studies are outlined in Table 1. The inclusion of non-wrist osteoarthritis or systemic conditions in the study sample was a common reason for excluding clinical trials that were identified in the searches. 

### 3.2. Patient-Reported Outcomes

A total of 9 condition-specific patient-reported outcomes were used across the included clinical trials (Table 2). The most common patient-reported outcome used in clinical trials for wrist osteoarthritis was the disabilities of the arm, shoulder, and hand questionnaire (DASH). The DASH was used in 4 of the 13 included clinical trials. The hand function visual analogue scale (VAS) was used in 3 clinical trials and the hand specific activities of daily living (ADL) questionnaire was used in 2 clinical trials. Six of the 9 condition-specific measures had been used in only one clinical trial. One trial did not implement a condition-specific patient-reported outcome in addition to other clinical measures such as pain, joint range of motion, and muscle strength. Satisfaction and appearance were each measured in one trial. Six instruments used a Likert scale for measurement; the remaining 3 instruments used a VAS.

A concise summary of manuscripts reporting the reliability, validity, and responsiveness of each of the 9 instruments is outlined in Table 3. Four out of the 9 instruments had empirical evidence reported for all elements of validity, reliability, and responsiveness under consideration in this paper. These were the DASH, MHQ, Patient Evaluation Measure (PEM), and Patient-Rated Wrist Evaluation (PRWE). Three instruments had no empirical studies reporting any components of validity, reliability, or responsiveness. Instruments with empirical evidence supporting content and construct validity included the DASH, MHQ, and PRWE. Instruments with empirical evidence supporting internal consistency, agreement, and reliability included the DASH and MHQ. Favourable responsiveness was also reported for the DASH, MHQ, PEM, and PRWE. The post-hoc review included 3 additional studies that did not meet inclusion criteria due to mixed rheumatoid arthritis and wrist osteoarthritis diagnoses samples and led to the identification of one additional outcome measure with favourable evidence supporting its validity, reliability, and responsiveness [42, 67–69]. This measure was the Australian/Canadian (AUSCAN) Osteoarthritis Hand Index [42, 67–69]. 

## 4. Discussion 

### 4.1. Main Finding

This investigation has identified 9 condition-specific patient-reported outcomes reported across 13 clinical trials amongst patients with wrist osteoarthritis (Table 1). Empirical evidence had been reported across all categories of validity, reliability, and responsiveness for four of these instruments (Table 2). The DASH and the MHQ had the most favourable and comprehensive supporting empirical evidence. The DASH was also the most commonly used condition-specific patient-reported outcome in the included clinical trials (Table 1). However, the PEM and PRWE also generally had favourable empirical evidence reported for elements of validity, reliability, and responsiveness. The AUSCAN Osteoarthritis Hand Index was also identified as a potentially useful instrument with favourable findings supporting its reliability, validity and responsiveness from a post-hoc review of studies excluded due to mixed rheumatoid and wrist osteoarthritis samples [42, 67–69]. 

Few investigations of validity, reliability, and responsiveness of these instruments included patients with osteoarthritis (Table 2). The findings reported in Table 3 should therefore be interpreted with caveats. These outcome measures have demonstrated reliability, validity, and responsiveness among mixed clinical population groups with diagnoses affecting the upper limb. An assertion that favourable measurement properties would hold true among patients with wrist osteoarthritis cannot be made with certainty. Nonetheless, in the absence of conflicting evidence, these empirical studies lend weight to the argument that these instruments are likely to have favourable measurement properties when used among people with wrist osteoarthritis participating in clinical trials. 

The selection of a patient-reported outcome for use in a clinical trial should be informed by several factors in addition to the measurement properties of the instrument [33]. Not all condition-specific patient-reported outcome measures are evaluating the same content or construct (Table 2). Similarly, not all instruments will have used the same response format (Table 2). These factors are important to consider when designing clinical trials. Other potential sources of bias beyond the scope of this paper may also influence the suitability of instruments for use in a particular clinical trial [31, 32, 70]. For this reason the summary information reported in this investigation is intended to be informative rather than provide prescriptive recommendations regarding which instruments should and should not be used. 

### 4.2. Comparison to Prior Research

The findings of favourable empirical psychometric data for the condition-specific patient-reported outcomes (Table 3) reported in this study are consistent with prior investigations of reliability, validity, and responsiveness of patient-reported outcomes amongst other orthopaedic patient groups [34, 40–42]. The post-hoc identification of the AUSCAN Osteoarthritis Hand Index as a relevant instrument with favourable psychometric properties is also consistent with prior reviews that included outcome measures for upper limb orthopaedic conditions [40–42]. This lends weight to the assertion that the AUSCAN Osteoarthritis Hand Index is also worthy of consideration for future clinical trials amongst people with wrist arthritis [42, 67, 68, 71–73]. 

### 4.3. Strengths and Limitations

The systematic audit trail approach to identify potential empirical evidence to support or refute the use of the patient-reported outcomes identified in this paper may be considered both a strength and weakness of this systematic review. A strength as it offers a systematic, albeit time consuming, method to identify studies that have reported elements of validity, reliability, and responsiveness. On the other hand, this citation trail audit would not have identified non-peer reviewed or unpublished sources of data reporting these attributes. However, this approach suited the aim of this investigation to review only published peer-reviewed reports of this information. 

A limitation to the extrapolation of study findings is that study designs other than randomised controlled trials were excluded. This approach was the most appropriate for addressing the research question at hand that dealt specifically with identifying instruments that have been used in clinical trial environments. However, instruments used in other contexts (such as population-based surveys) were not included in the scope of this review. 

### 4.4. Future Research

Findings from this investigation have several key implications for future research. Future clinical trials should consider the reliability, validity, and responsiveness of patient-reported outcomes under consideration for inclusion in clinical trial assessments. The DASH, MHQ, PEM, PRWE, and AUSCAN Osteoarthritis Hand Index all have published peer-reviewed empirical evidence available to inform this decision. Future research involving the other instruments reported in this investigation (Table 2) should consider exploring the properties (validity, reliability, and responsiveness) not yet reported. Additionally, it would be useful for future investigations involving wrist arthritis populations to consider empirical work confirming or refuting the measurement properties of the instruments included in this paper with data obtained from patients suffering from wrist arthritis. 

## 5. Conclusions 

This investigation identified a range of condition-specific patient-reported outcome measures that have been used in clinical trials amongst patients with wrist osteoarthritis. The DASH was the most commonly used instrument across these clinical trials. The DASH and MHQ both had consistent favourable findings across all elements of validity, reliability, and responsiveness under consideration. However, the PEM and PRWE also generally had favourable empirical evidence reported for elements of validity, reliability, and responsiveness. The AUSCAN Osteoarthritis Hand Index was not used among trials meeting the strict inclusion criteria. However, it was identified as another suitable instrument with favourable reliability, validity, and responsiveness and is also worthy of consideration for future clinical trials among people with wrist osteoarthritis. 

## Figures and Tables

**Table 1:** Summary of the sample, intervention, and outcome measures used in included studies.

Author (year)	Clinical population	Intervention	Patient-reported outcomes	Other measures
Nilsson et al. [43]	109 adults with painful carpometacarpal OA*	Surgical implantation of Artelon carpometacarpal joint spacer	Pain VAS* DASH* questionnaire	Strength: lateral and three-finger pinch ROM: thumb abduction

Bisneto et al. [44]	20 adults with wrist OA, with a diagnosis of scapholunate advanced collapse (*n* = 4) or scaphoid nonunion advanced collapse (*n* = 16)	Proximal row carpectomy or four-corner fusion	DASH questionnaire Pain VAS	ROM*: wrist flexion/extension, radial/ulnar deviation, pronation/supination Strength: grip, pulp-pulp, lateral, and three-finger pinch Two-point discrimination: 2nd and 5th fingers, dorsum of 1st web Hand and wrist volume Jebson-Taylor functional test

Ritchie and Belcher [45]	41 adults with OA of the trapeziometacarpal joint	Trapeziectomy (anterior or posterior approach)	ADL* questionnaire (10 items relating to hand function) Hand function VAS Thumb pain VAS Power VAS Range of motion VAS Cosmesis VAS	ROM: thumb joint including opposition, palmar abduction, and extension of trapeziometacarpal joint Strength: grip, thumb, and three-finger pinch Radiograph Scar tenderness

Belcher and Nicholl [46]	36 adults (42 hands) with OA of the trapeziometacarpal joint	Trapeziectomy (with/without ligament reconstruction and tendon interposition)	ADL* questionnaire (10-items relating to hand function) Hand function VAS Thumb pain VAS Satisfaction with surgery VAS	ROM: thumb interphalangeal, metacarpophalangeal and trapeziometacarpal joints Strength: grip, thumb and three finger pinch Radiograph

Horlock and Belcher [47]	39 adults (40 hands) with OA of the 1st carpometacarpal joint	Early versus late mobilisation following simple trapeziectomy	Hand function VAS Thumb pain VAS	ROM: interphalangeal, metacarpophalangeal and first carpometacarpal joints Strength: grip, thumb and three finger pinch Radiograph

Jain et al. [48]	62 adults (84 joints) with painful OA of the trapeziometacarpal joint	Transdermal steroids	Michigan Hand Outcomes Questionnaire (MHQ) Short form 12 (SF12) Pain VAS	ROM: thumb hyperextension Thumb adduction contractures Strength: grip, tip-to-tip, and lateral pinch

Fuchs et al. [49]	56 adults with OA of the carpometacarpal joint	Intra-articular injection (sodium hyaluronate)	Pain VAS	Heat, swelling, and crepitations under palpation Strength: lateral and pulp pinch ROM: radial and palmar abduction/adduction, opposition

Davis et al. [50]	162 women (183 thumbs) with OA of the trapeziometacarpal joint	Trapeziectomy	—	Subjective measures of: thumb pain, stiffness, and restriction of ADLs Strength: grip, tip-to-tip and lateral pinch ROM: thumb joint, palmar and radial abduction, opposition, and hyperextension

Kriegs-Au et al. [51]	43 adults (53 thumbs) with OA of the thumb carpometacarpal joint	Trapeziectomy with ligament reconstruction (with/without tendon interposition)	Questionnaires relating to pain, strength, daily function, dexterity, cosmetic appearance, willingness to undergo similar surgery, and satisfaction with surgery 10-item questionnaire relating to functional tasks	Buck-Gramcko score ROM: Palmar and radial abduction, opposition, and hyperextension Strength: grip and tip pinch Radiograph

Weiss et al. [52]	25 adults with OA of the carpometacarpal joint	Custom-made short opponens thermoplastic or prefabricated short neoprene splints	Pain severity/duration VAS Tip pinch pain VAS 22-item ADLs questionnaire Splint rating VAS	Strength: Tip pinch Radiograph

Field et al. [53]	65 adults with OA of the carpometacarpal joint	Trapeziectomy (with or without flexor carpi radialis suspension)	Pain VAS	ROM: thumb joint, radial, and palmer abduction, 1st web space span Strength: grip, three-finger and, tip pinch Radiograph Procedure satisfaction

De Smet et al. [54]	56 females with painful OA of the 1st carpometacarpal joint	Trapeziectomy (with or without tendon interposition/ligament reconstruction)	Pain VAS Pain relief questionnaire DASH questionnaire Functional outcome questionnaire	ROM: all thumb joints, web angle Strength: grip and three-finger pinch Radiograph

Davis and Pace [55]	113 adults (133 thumbs) with OA of the trapeziometacarpal joint	Trapeziectomy: with ligament reconstruction, tendon interposition, and Kirschner wire insertion followed by splintage or with no Kirschner wire and immobilisation in a soft bandage	Patient Evaluation Measure (PEM) DASH questionnaire	Thumb pain, strength, and stiffness ROM: trapeziometacarpal joint extension, palmar abduction, opposition, and metacarpophalangeal hyperextension Strength: grip, three-finger, and tip pinch

*Abbreviations: OA: osteoarthritis, DASH: disabilities of the arm, shoulder and hand questionnaire, VAS: visual analogue scale, ROM: range of motion.

**Table 2:** Characteristics of identified condition (or body region) specific patient reported outcomes.

Measure^*^*^	Identified studies citing the measure	Primary reference for measure	Number of unique citations for the measure	Anatomical region	Assesses	Number of items (type)
Cosmesis visual analog scale (VAS)	[45]	None^†^	None^†^	Hand	Appearance	1 (100 mm VAS)
Disabilities of the arm, shoulder, and hand (DASH)	[43, 44, 54, 55]	[56]	1103	Upper limb	Symptoms, function	30 (5-point Likert)
Hand function visual analog scale (VAS)	[45–47]	None^†^	None^†^	Hand	Function	1 (100 mm VAS)
Hand specific ADL questionnaire	[45, 46]	[57]	18	Hand	Function	10 (4-Point Likert)
Michigan Hand Outcomes Questionnaire (MHQ)	[48]	[58]	196	Hand	Symptom, function, satisfaction	37 “core” items (5-point Likert)
Patient Evaluation Measure (PEM)	[55]	[59]	71	Hand/wrist	Symptom, function	10 (7-point Likert)*
Patient-Rated Wrist Evaluation (PRWE)	[60]	[61, 62]	210	Wrist/hand	Symptom, function	15 (11-point Likert)
Perceived grip strength scale	[53]	None^†^	None^†^	Hand	Function	1 (100 mm VAS)
Scale of hand-specific activity performance	[51]	None^†^	None^†^	Hand	Function	10 (5-point Likert)

^*^*^Each measure is patient-administered.

*The total measure is comprised of 18 items; 10 of which form the Hand Health Profile.

^†^Trial did not cite a source for this measure. No article cited the trial-reported reliability, validity, or responsiveness of the instrument.

**Table 3:** Summary of quality ratings for identified measures.

Measure	Content validity	Construct validity	Internal consistency	Interrater agreement	Reliability	Responsiveness
Cosmesis visual analog scale (VAS)	?	?	?	?	?	?
Disabilities of the arm, shoulder, and hand questionnaire (DASH)	+	+	+	+	+	+
Hand function visual analog scale (VAS)	?	?	?	?	?	?
Hand-specific ADL questionnaire	?	0	0	?	?	?
Michigan Hand Outcomes Questionnaire (MHQ)	+	+	+	+	+	+
Patient Evaluation Measure (PEM)	0	+	+	0	+	+
Patient-Rated Wrist Evaluation	+	+	+	0	+	+
Perceived grip strength scale	?	?	?	?	?	?
Scale of hand-specific activity performance	?	?	?	?	?	?

+: positive rating; 0: substantially conflicting results or methodology concerns (including unclear methodology description); −: negative rating (not required); ?: no/insufficient information.

Note: Psychometric testing of these measures among any clinical population with upper limb pathology was included.
